# Pancreatic Cysts

**Published:** 1899-03-25

**Authors:** 


### PANCREATIC CYSTS.

As the result of the discussion on a case of pancreatic cyst treated by incision and drainage brought before the Clinical Society by Mr. Arthur Barker, it seems pretty clear that this treatment is the proper one to adopt in such cases, enucleation being too great a risk. Mr. Barker opened the abdomen by a three-inch vertical incision through the left rectus muscle, close below the ribs. On opening the peritoneum the stomach was found to be stretched over the tumour, and the transverse colon lay below it. Between the two there was a small space through which the cyst was tapped, giving issue to three or four pints of fluid, at first clear and light coloured, but becoming slightly brown towards the end. He sutured the lips of the cyst to the edges of the wound, and left in an iodoform gauze drain. The patient did well.

